# Genetic erosion within the Fabada dry bean market class revealed by high‐throughput genotyping

**DOI:** 10.1002/tpg2.20379

**Published:** 2023-09-19

**Authors:** Maria Jurado, Carmen García‐Fernández, Ana Campa, Juan Jose Ferreira

**Affiliations:** ^1^ Plant Genetic Group Regional Service for Agrofood Research and Development (SERIDA) Villaviciosa Spain

## Abstract

The Fabada market class within the dry beans has a well‐differentiated seed phenotype with very large white seeds. This work investigated the genetic diversity maintained in the seed collections within this market class and possible genetic erosion over the last 30 years. A panel with 100 accessions was maintained in seed collections for 30 years, 57 accessions collected from farmers in 2021, six cultivars developed in SERIDA, and 16 reference cultivars were gathered and genotyped with 108,585 SNPs using the genotyping‐by‐sequencing method. Filtering based on genotypic and phenotypic data was carried out in a staggered way to investigate the genetic diversity among populations. The dendrogram generated from genotyping revealed 90 lines forming 16 groups with identical SNP profiles (redundant lines) from 159 lines classified as market‐class Fabada according to their passport data. Seed phenotyping indicated that 19 lines were mistakenly classified as Fabada (homonymies), which was confirmed in the dendrogram built without redundant lines. Moreover, this study provides evidence of genetic erosion between the population preserved for 30 years and the currently cultivated population. The conserved population contains 54.6% segregation sites and 41 different SNP profiles, whereas the cultivated population has 19.6% segregation sites and 26 SNP profiles. The loss of genetic variability cannot be attributed to the diffusion of modern cultivars, which increase genetic diversity (six new SNP profiles). The results allow for the more efficient preservation of plant genetic resources in genebanks, minimizing redundant accessions and incorporating new variations based on genotypic and phenotypic data.

## Core Ideas

- High‐throughput genotyping based on GBS revealed wide variation in the Fabada market class.
- Phenotyping plus genotyping revealed redundant and mistakenly classified lines.
- Genetic erosion was observed between the conserved in ex situ collections and cultivated accessions.
- Genetic erosion cannot be attributed to the spread of modern cultivars.

**Abbreviations**
GBS: genotyping by sequencing
MAF: minor allele frequency
PCoA: principal coordinate analyses
QTL: quantitative trait locus
SDP: Spanish diversity panel
SERIDA: Regional Service for Agrofood Research and Development
SNP: single‐nucleotide polymorphism

## 1 INTRODUCTION

The common bean (*Phaseolus vulgaris* L.) is the second most important cultivated legume species in the world (33 million ha; FAO, 2020) and the most important legume for human consumption. The common bean is a diploid (2*n* = 2*x* = 22) and self‐pollinated species domesticated by Mesoamerican and Andean cultures. Two gene pools corresponding to those geographical regions have been reported for both wild and cultivated common beans based on morphological traits, seed proteins, and molecular markers variation (Kwak & Gepts, 2009; Rodriguez et al., 2016; Singh et al., 1991). From these regions, the species was progressively dispersed worldwide (Angioi et al., 2010; Asfaw et al., 2009; Gepts et al., 1986; Zhang et al., 2008). In Europe, local germplasms from both gene pools are present, as well as intermediate genotypes are probably derived from recombination between the two gene pools (Angioi et al., 2010; Bellucci et al., 2023; Campa et al., 2018; Santalla et al., 2002).

Bean seeds exhibit wide phenotypic diversity, including size, shape, and coat‐color variations (Singh, 1989; Voysest, 2000). Seed coat colors vary widely (white, cream, yellow, brown, red, purple, and black) and have different intensities, as well as patterns that combine different colors (e.g., bicolor, mottled, and spotted). Cultivars with 20–100 g/100 seed‐weights have been observed (Voysest, 2000). Dry beans have been grouped into differentiated seed‐based phenotypic groups or market classes, such as Navy, White Kidney, Great Northern, Canellini, Fabada, Yellow, Carioca, Small Red, Red Mexican, Red Kidney, Cranberry, Pinto, and Black. Among them, market‐class Fabada (syn. Faba granja and favada) has a well‐differentiated seed phenotype in the species with very large white seeds (∼100 g/100 seeds) having an oblong shape with a length/width ratio greater than 2.2. Fabada had already been described in the north of Spain by the mid‐20th century (Romero, 1961). In the last 30 years, the Fabada crop has undergone notable changes: transition from bean‐maize intercropping to monoculture, expansion of cultivation to new areas, diversification of uses, emergence of new cultivars from breeding programs, and modernization of farming methods. Landraces have climbing indeterminate growth habits, whereas modern cultivars can have both determinate and climbing indeterminate growth habits (Ferreira et al., 2017). Previous analyses indicated that Fabada landraces were not a homogeneous group. Santalla et al. (2002) reported variations in this market class based on allozyme and phaseolin polymorphisms. Three genotypes of this market class (cv. Andecha, Maruxina, and Xana) were included in the Spanish diversity panel (SDP), and they were not grouped, with one of the two main gene pools showing an intermediate position (Campa et al., 2018).

Measures of genetic diversity in cultivated plants and their wild relatives are needed to make decisions, monitor changes, and warn of emerging problems in agricultural production (Brown & Hodgkin, 2015). Most diversity studies in common beans have focused on a wide range of phenotypes from many places or maintained in wide seed collections (Angioi et al., 2010; Campa et al., 2018). The classification of bean‐seed phenotypic diversity based on market classes is useful to describe the diversity or variation as a first approach, but within the same market class, there may be different levels of phenotypic and genotypic variation. Moreover, there are seed phenotypes that hardly fit the described market classes. A detailed characterization of variation in the market classes will help to efficiently preserve genetic diversity and to better differentiate and identify prominent genotypes. For diversity preservation, it is relevant to know the amount of variation to efficiently maintain and use diversity. Additionally, the changes in the diversity cultivated over time and possible genetic erosion provide interesting information on the diversity preserved in the ex situ collections. The literature reports cases of lost genetic diversity in cultivated species and landraces over the past century and continuing into the present (Khoury et al., 2022). Crop erosion can occur at the level of crop species, variety, or allele. Genetic erosion, understood as a reduction in allelic evenness and richness, is directly related to the breeding capabilities, vulnerability, evolutionary potential, and resilience of crops (Brown & Hodgkin, 2015; Fu, 2015; van de Wouw et al., 2010). Crop genetic diversity has traditionally been analyzed using morphological traits. However, genetic diversity changes within a varietal type are more difficult to document owing to the limited number of phenotypic markers. At present, high‐throughput genotyping methods (e.g., genotyping by sequencing [GBS] method; Elshire et al., 2011) identify many markers per genotype in comparison to a reference genome and provide a picture of the genetic diversity landscape.

**Core Ideas**

- High‐throughput genotyping based on GBS revealed wide variation in the Fabada market class.
- Phenotyping plus genotyping revealed redundant and mistakenly classified lines.
- Genetic erosion was observed between the conserved in ex situ collections and cultivated accessions.
- Genetic erosion cannot be attributed to the spread of modern cultivars.

A large collection of landraces representing the Spanish diversity of the Fabada market class is maintained in the National Collection of Plant Genetics (CRF‐INIA‐CSIC, Madrid; https://bancocrf.inia.es/es/). Most of these accessions were collected before 1991, and a duplicate collection has been maintained in the Regional Service for Agrofood Research and Development (SERIDA) collection for 30 years. On the other hand, new cultivars of the Fabada market class have been produced by different plant breeding programs (Ferreira et al., 2012) and disseminated over the last 20 years, such as cv. Andecha, Maruxina, Maximina, and Xana. Thus, there is an opportunity to investigate the genetic erosion within this market class, which could be extrapolated to global conditions in which the rapid modernization of crops has occurred. This work investigated the genetic diversity of landraces grouped in the market class Fabada and the possible loss of genetic diversity in the currently cultivated material. The results are relevant for the efficient preservation of species diversity as well as the suitable use of genetic diversity in this market class.

## 2 MATERIALS AND METHODS

### 2.1 Plant material

**FIGURE 1:**
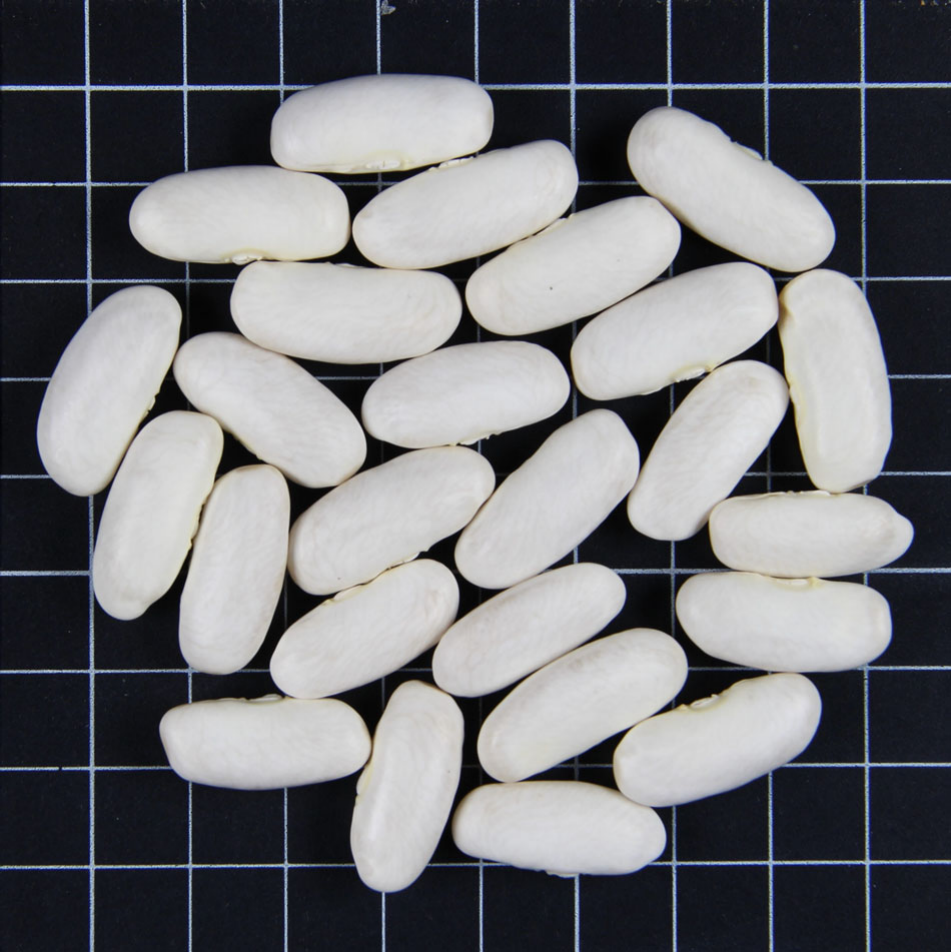
Seed phenotype of the Fabada market class (syn. Faba granja and favada). The bars represent 1 cm.

A total of 179 *P. vulgaris* accessions were gathered into a panel (FabaPanel). Table S1 presents the list of materials included in this study as well as the respective passport data. The FabaPanel contains 100 accessions conserved in the SERIDA seed collection and recorded as Fabada market class in respective passport data (conserved population; code FP032 to FP456). Most of these Fabada accessions were collected in Northern Spain before the 1990s. Moreover, 57 Fabada accessions with indeterminate growth habits were collected in Northern Spain from local farmers in 2021, and they were also included in the FabaPanel (cultivated population: FP500 to FP559). Six breeding lines developed in SERIDA having the Fabada seed phenotype were added as a control (cultivar population): A25 (cv. Andecha, an old cultivar marketed since the early 2000s; Figure 1), and the lines B8, Xana, A2806 (cv. Maximina, distributed since the early 2010s), X4562, and A4804. In addition, 16 well‐known genotypes were added to this panel as a reference diversity population: AB136, BAT93, Cornell49242, Cannellini, DOR364, Musica, G19833, Garrafal Oro, La Victorie, IVT7214, MDRK, Planeta, SanilacBc6Are, Tendergreen, TU, and Midas (reference population). Those reference genotypes are also included in the SDP (Campa et al., 2018).

A homozygous line per accession was obtained by self‐pollinating individual plants derived from each accession. The crop was developed in a greenhouse, and it was used to collect the tissues for genotyping and phenotyping of the harvested seeds. Seed phenotyping was based on their visual characteristics, including color, size, and shape.

### 2.2 DNA isolation and genotyping

Genomic DNA was isolated from young leaf samples using the SILEX method (Vilanova et al., 2020), and DNA quality was checked in agarose gels. The GBS method was carried out following Elshire et al. (2011) and optimized by Schröder et al. (2016) using the *Taq*αI and *Mse*I restriction enzymes. Library construction was performed following the protocol of Poland et al. (2012) with modifications in the adaptors for ligation. In total, 20 barcoded samples were pooled for PCR amplification. Sequencing was performed in the Illumina platform by Macrogen Inc. Single‐nucleotide polymorphism (SNP) calling was carried out by AllGenetics&Biology SL (www.allgenetics.eu) using the reference genome of *P. vulgaris* (Pvulgaris_442_v2.0) obtained from the JGI Data Portal (https://phytozome‐next.jgi.doe.gov/info/Pvulgaris_v2_1).

### 2.3 Panel filtering

A stepped filtering method was developed to identify redundant or off‐type lines. Genotyping data of the FabaPanel were filtered with the help of the software Tassel v5 (Bradbury et al., 2007). First, the homozygous genotypes with more than 50% missing data and SNPs located out of the 11 bean pseudo‐chromosomes were removed from the FabaPanel. Then, the constituted subsets of the FabaPanel were filtered using the following criteria: the proportion of missing data (<10%) and minor allele frequency (MAF > 0.05 when the reference genotypes were included; MAF > 0.01 when the reference genotypes were not included). Finally, the lines classified as Fabada according to their passport data but having seed phenotypes that did not correspond to this market class were removed.

### 2.4 Population diversity and clustering analyses

The R package SambaR_v1.08 (de Jong et al., 2021) was used to import raw data files into R and perform the diversity analysis. Principal coordinate analyses (PCoA) based on Hamming's distance were conducted using the function ape_pcoa(). Population differentiation measures for all pairwise population comparisons (Fst and Nei's distance) were performed with the function calcdistance().

Dendrograms were built from Euclidean distance using the unweighted pair group method with arithmetic mean method for clustering analysis with the help of the packages “ggplot2” (Wickham, 2016), “FactoMinerR” (Lê et al., 2008), “factoextra” (Kassambara & Mundt, 2020), “cluster” (Maechler et al., 2022), and “ape” (Paradis & Schliep, 2019) in R software (R Core Team, 2022).

The numbers of segregating sites (SNP) and SNP profiles per population were used to estimate the genetic diversity and putative genetic erosion. An SNP profile is defined by the same genotype for all SNPs. The number of segregating sites per population was obtained with the help of the software Tassel v5. The number of SNP profiles was obtained from the dendrogram constructed after filtering the populations. Finally, the diversity per population was estimated using the Shannon Diversity Index [H’ = −Σ*pi* * ln(*pi*)], where *p_i_* represents the proportion of the SNP profile *i*. The Shannon equitability index (which measures the evenness of profiles in a population) was also estimated as E_H_ = *H*′/ln(*S*), where *H*′ and *S* represent the Shannon index and the number of SNP profiles, respectively.

## 3 RESULTS

### 3.1 Genotyping of the FabaPanel

**FIGURE 2:**
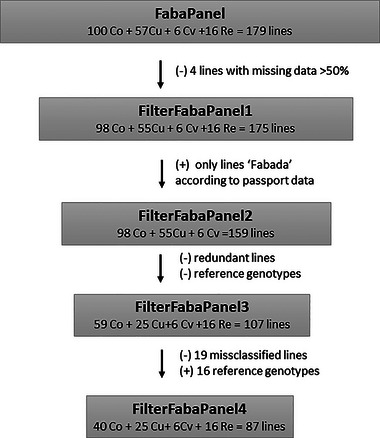
Established subsets from FabaPanel to investigate changes in diversity in the Fabada market class. The composition of each subset and the filtering criteria are also indicated. The lines included in each subset are described in Table S1. Co, conserved population; Cu, cultivated population; Cv, cultivars; Re, reference population.

Sequencing of the GBS libraries yielded approximately 90.7 million reads per line (an average of 816.4 million reads per library for 20 lines), and the Q20 value of each library was greater than 97.24%. The GBS analysis generated 108,585 SNPs. Based on this genotyping, a staggered filtering process was carried out to specifically study the diversity of the Fabada market class (Figure 2). First, four lines showed missing data for more than 50% of the obtained SNPs (FP173, FP174, FP508, and F541) and were therefore removed (FilterFabaPanel1). The genotyping of the remaining 175 lines was filtered considering homozygous sites, location in one of the 11 bean chromosomes, missing values (<10%), and MAF (>0.05), resulting in 22,259 SNPs. The number of SNPs per chromosome ranged from 1214 for chromosome Pv06 to 3088 for chromosome Pv11 (Figure S1). After filtering and thinning the data, the mean proportion of missing data per individual was 3%, the GC content was 0.5, and the transition versus transversion ratio was 2.28. The most frequent transition and transversion events were A/G and C/T, respectively.

### 3.2 Filtered from genotyping

Table S1 presents the lines included in the subsets constituted to develop this study. A subset, named FilterFabaPanel2, was specifically designed from FilterFabaPanel1 to investigate the redundancies in the market class “Fabada” (see Figure 2). This FilterFabaPanel2 had 159 lines, all of them recorded as “Fabada” in the respective passport data (or Faba granja), and was genotyped with 21,837 SNP after filtering (MAF > 0.01): 98 lines from the SERIDA collection (conserved population), 55 lines collected in 2021 (cultivated population), and six breeding lines (cultivar population; see Table S1). A PCoA based on Hamming's distance showed two main coordinates that explained a total of 93.9% of the variance (69.2% and 24.7%, respectively). The scatter plot built with these two main components (Figure S2) exhibited a higher dispersion in the lines included in the conserved population than in the other two populations, the cultivar and cultivated populations. The plot also revealed many overlapping lines. In parallel, a dendrogram was constructed (Figure S3), showing that 90 lines were grouped in 16 groups with more than one line with identical SNP profiles (41% of redundant lines or duplicate accessions). For instance, it was the case of the lines maintained in the SERIDA collection: FP102, FP103, FP106, FP108, FP115, FP119, FP133, FP147, FP148, FP154, and FP182. Additionally, 69 lines had unique SNP profiles, so 85 SNP profiles were detected for FilterFabaPanel2 (69 unique + 16 non‐unique). However, we also observed closely related lines that differ by a few SNP; for example, the breeding line A2806 and the group with the lines FP501, FP524, and FP528 differ by two SNP, and the line A25 and the group with the lines FP502, FP540, and FP559 also differ by two SNP. Finally, the dendrogram also indicated that most of the lines collected in 2021 (cultivated population) were closely related and grouped together the line A25.

**FIGURE 3:**
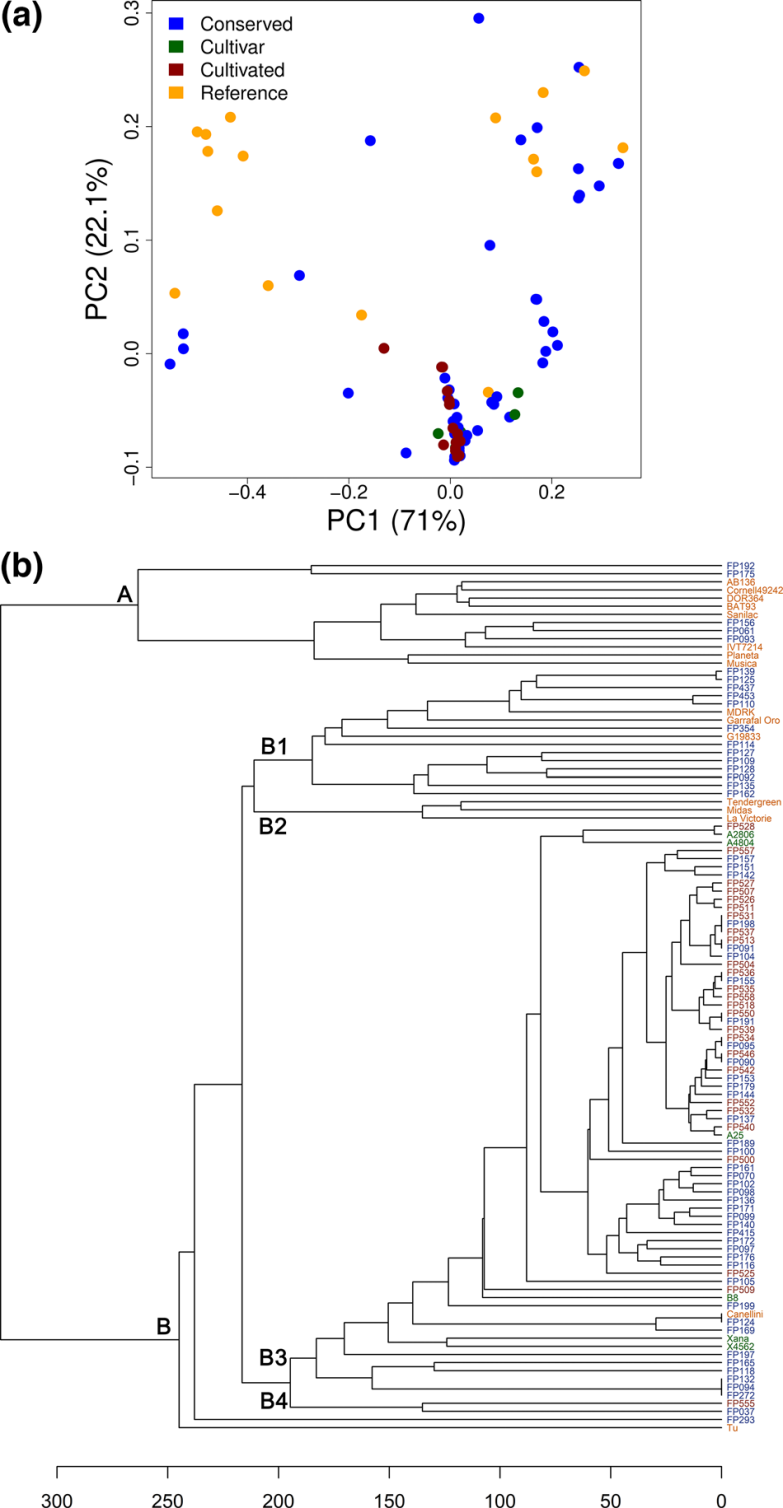
(a) Scatter plot obtained with the two mains coordinates revealed by the principal coordinate analysis (PCoA) based on Hamming's distance of the 107 lines genotyped with 21,618 single‐nucleotide polymorphisms (SNPs). (b) Dendrogram generated from 107 lines genotyped with 21,618 SNPs using the Euclidea distance and the unweighted pair group method with arithmetic mean clustering method (FilterFabaPanel3).

Redundant lines in FilterFabaPanel2 from the first generated dendrogram were removed, maintaining one genotype per SNP profile and population (conserved, cultivated, and cultivars). The resulting set contained 91 lines (Table S1) that were used to build a new subset along with the 16 lines from the reference population (FilterFabaPanel3, 107 lines). The PCoA of FilterFabaPanel3 revealed two main coordinates explaining 93.1% of the variance, and the generated plot showed wide dispersions for the conserved and reference populations (Figure 3a). In contrast, the lines of the cultivar and the cultivated population were less dispersed. As shown in Figure 3b, the dendrogram has two main branches. The “A” group contained 13 lines, including typical Mesoamerican cultivars such as Sanilac, Cornell49242, and AB136. Group “A” included five lines from the conserved population (FP192, FP175, FP156, FP061, FP093), which were all recorded as Fabada market classes in their respective passport data. The “B” group contained 91 lines, including typical Andean cultivars such as MDRK, Tendergreen, and G19833. Most of the lines classified as the market class Fabada were classified as Group B, and the following four subgroups were established:

1. Group B1 is formed by 16 lines, including FP139, FP125, FP437, FP453, FP110, MDRK, Garrafal Oro, FP354, and G19833, one of the bean genomes available (Schmutz et al., 2014).
2. Group B2 is formed by three snap bean cultivars: Tendergreen, Midas, and La Victorie.
3. Group B3 consisted of 71 lines that included the six cultivars of the Fabada market class, 39 lines from the conserved population, and 25 lines from the collected population. The reference cultivar Canellini was included in this group and was close to the cultivars Xana and X4562, which both have determinate growth habits.
4. Group B4 is formed by the remaining two lines, FP037 and FP555.

Finally, the lines TU and FP293 were located far from the four B subgroups described above.

### 3.3 Filtered from phenotyping

The harvested seeds from self‐pollinated plants in the greenhouse were phenotyped by considering color, size, and shape. The phenotyping indicated that 19 lines registered as Fabada market class in their respective passport data did not have the full characteristics of the market class Fabada (homonymy; the same local name but different seed phenotype): FP037, FP061, FP093, FP110, FP114, FP118, FP124, FP125, FP128, FP135, FP139, FP156, FP165, FP175, FP192, FP293, FP354, FP437, and FP453 (see images at https://zenodo.org/record/7015279#.Y1veBORByUk; Table S1). These lines had white and oblong seeds, but they were of a smaller size than those of the Fabada market class. These 19 lines were located in the dendrogram outside the main group containing cultivar A25 (Group B3). The lines FP092, FP094, and FP272 were difficult to classify because their seed phenotypes were similar to the Fabada market class, with seed size being intermediate between the large Canellini and the Fabada market class.

### 3.4 Genetic diversity in the Fabada market class

To investigate the specific genetic diversity in the Fabada market class, a subset (FilterFabaPanel4) of the FabaPanel was created after removing redundant genotypes and misclassifications in the conserved population, which had been revealed by the previous analysis. This subset of FabaPanel had 88 lines genotyped with 21,618 SNPs after filtering (missing values <10% and MAF > 0.01), and it contained the six cultivars: 26 lines from the cultivated population, 40 lines from the conserved population, and 16 lines from the reference population. The distribution of the segregating sites per population is shown in Figure S5, and the percentages of segregating sites (SNPs) per chromosome in the four populations are shown in Table 1. The highest percentage was observed in the reference population (mean 99.68%), followed by the conserved population (54.56%). The cultivated population exhibited lower percentages of segregating sites than the conserved population for all the chromosomes except chromosome Pv11. The cultivar population had a higher percentage of chromosome Pv01 than the cultivated and conserved populations. Very high polymorphism levels (>90%) were found in the conserved population for chromosomes Pv04, Pv05, and Pv08. The estimations of the diversity indices using the SNP profiles showed a higher Shannon diversity index (H’) for the conserved population (Table 1), whereas the lowest value was observed in the cultivar population. This population contained only six breeding lines, all with different SNP profiles. Among the four populations, the fixation index (Fst) ranged from 0.018 (cultivar and conserved populations) to 0.329 (reference and cultivated populations), whereas the Nei´s genetic distance varied between 0.028 (cultivated and cultivar populations) and 0.31 (reference and cultivated populations; Table S2).

**TABLE 1:** Diversity assessments in the FilterFabaPanel4.

		Segregating sites (%) per population			
Chr	No. SNP	Cultivars	Cultivated	Conserved	Reference
**Pv01**	2265	40.75	3.44	9.62	99.91
**Pv02**	1473	3.46	19.42	69.31	99.32
**Pv03**	2237	29.50	5.19	72.69	99.82
**Pv04**	2512	62.90	25.48	91.76	99.84
**Pv05**	2058	48.88	42.66	94.46	99.90
**Pv06**	1171	65.16	4.18	79.68	99.91
**Pv07**	1465	11.54	2.87	51.88	99.25
**Pv08**	2630	5.63	26.46	92.55	99.96
**Pv09**	1410	1.56	1.56	9.08	99.79
**Pv10**	1515	6.01	0.46	3.96	99.80
**Pv11**	2882	20.92	84.25	25.16	98.99
**Mean**		26.94	19.63	54.56	99.68
**S**		6	25	40	16
**H’**		0.17	1.79	2.64	0.47
**E_H_**		0.09	0.38	0.49	0.17

*Note*: The lines included in this subset are described in Table S1. Percentage of segregating sites (single‐nucleotide polymorphism [SNP]) per chromosome in the four populations considered: conserved, cultivated, cultivar, and reference.

Abbreviations: S, richness of genotypes (SNP profiles); H’, Shannon diversity index; E_H,_ Shannon equitability index.

A map with the segregating sites along the bean genome showed relevant differences among the Fabada lines compared with the A25 line (Figure S5). For example, the lines FP555 and FP509 show SNPs located on chromosome Pv11. The determinate cultivars Xana and X4562 present many SNPs located at the end of Pv01, where the gene *fin* (introgressed in these lines) is located. The lines FP094, FP132, and FP272 were noted for localized variations along chromosome Pv08, whereas lines FP092, FP109, FP137, and FP162 were noted for localized variations along chromosome Pv04. Lines FP555 and FP197 showed specific variations in Pv05, and lines A2806, A4804, X4504, and FP528 exhibited very specific variations at the end of chromosome Pv11, in the area of the Co‐2 anthracnose resistance cluster.

## 4 DISCUSSION

In this study, the variation within the Fabada dry bean group was investigated for efficient preservation and use of the genetic diversity in this market class. This market class has a well‐differentiated seed phenotype and high‐quality seeds. A panel with 179 accessions was gathered, and the lines were classified into four groups based on their origin: conserved, cultivated, cultivar, and reference populations. The genotyping of the panel yielded a high number of SNPs (108,585) that homogeneously covered the 11 bean chromosomes (Figure S1). Filtering based on genotyping and phenotyping data was carried out to fine‐tune the estimation of diversity in the Fabada market class. Genotypic variation was first detected in redundant lines; 16 groups consisted of more than one line that did not contain differences in the SNP genotypes. These 16 groups included 90 lines (52 from conserved and 38 from cultivated populations), resulting in 41.8% redundancy in the conserved population (41 of 98) and 50.9% in the cultivated population (28 of 55). Seven of those 16 groups included lines of both the conserved and cultivated populations; consequently, most of the genetic diversity in the cultivated population was already present in the SERIDA collection. In contrast, the lines FP555, FP509, FP525, FP500, FP552, and FP542, which have different SNP‐related genotypes from the lines of the conserved populations, represent a source of variation not maintained in the SERIDA collection. These findings can be used to optimize preserving the collection by reducing the accessions conserved in lines having identical SNP profiles and incorporating those lines that were different from the cultivated population. Likewise, the variation observed within this market class agreed with the hypothesis that it is a landrace (Zeven, 1998) because if it was produced by a breeding program, a lower variation would be expected. The clustering also showed six lines that were very closely related (different for only two to four SNPs) to the old cultivar A25 (F559, FP502, and FP540) and the modern cultivars A2806 (FP528, FP501, and FP524), indicating that they probably are derived from these cultivars because it is very common for local farmers to use harvested seed for planting.

The filtering carried out with genotyping data in the conserved and cultivated populations reduced the FabaPanel to 107 lines, removing redundant lines and maintaining a line per population (SNP profile). The PCoA plot and the generated cluster indicated the wide diversity of lines classified as Fabada market class. However, the phenotypic characterization of seeds indicated that 19 lines had seeds with characteristics that differed from those of the Fabada market class (homonymy). These lines were located far from the A25 line in the generated dendrogram. Seed size is a quantitative inheritance trait with moderate heritability, and there was an environmental effect on the expression of the characteristic (Murube et al., 2020). In addition, there is an overlap in seed size between the large Canellini or White Kidney (seed length 17–20 mm) and Fabada (seed length 20–24 mm) market classes that can induce errors in the classifications. In fact, a Cannellini line in the reference population shows an SNP profile that is identical to that of line FP124 and is similar to that of FP169. The Fabada cultivars Xana and X4562, both with determinate growth habits derived from V203, are very close to Canellini accessions maintained in the SERIDA collection (Ferreira et al., 2017).

To study the putative genetic erosion into the Fabada market class in the past 30 years, those redundant lines and the misclassified lines were removed from FabaPanel. The diversity valued as the percentage of segregating sites indicated an important genetic erosion, a loss of genetic diversity between the cultivated and the conserved populations (19.62%–54.26%; Table 1). The changes are also reflected in the loss of SNP profiles and the Shannon diversity index; for example, the conserved population had 40 different profiles of SNPs (40 of 84, 47.6%), while the cultivated population had 26 profiles (30.9%), many of them common to the conserved population (6). Crop genetic erosion has been attributed to anthropogenic and environmental factors (Khoury et al., 2022; van de Wouw et al., 2010), such as the replacement of landraces by modern cultivars. Two Fabada commercial cultivars distributed more than 10 years ago were included in this study: A25 and A2806. However, the genetic erosion cannot attribute to diffusion of new cultivars because only six of the 57 lines of the cultivated population can be considered to be derived from commercial cultivars (10.2%). Rather, this genetic erosion may be due to the reduction in the number of farmers in the last 30 years, the selection of sowing seeds, and the increase of monocropping against the traditional maize‐bean intercropping. Local farmers usually use their sowing seeds in plantings and select the largest seed to be used in the next planting. This custom explains the limited dissemination of modern varieties and is a driver of selection of seed phenotypes.

The genomic exploration of segregation sites along the 11 bean chromosomes showed chromosomal regions with high and no variation (no SNPs; Figures S4 and S5). The six cultivars included in this study exhibited higher diversity levels than the members of the cultivated population owing to the introgression of new genomic regions in chromosomes Pv01, Pv02, Pv04, Pv06, and Pv11. The lines Xana, X4562, A2806, and A4804 were originally derived from line A25 (Ferreira et al., 2017), and they carry new genes on chromosomes Pv01 (gene *fin*), Pv02 (gene *I*), Pv04 (gene *Pm1*), and Pv11 (gene *Co‐2*). Major quantitative trait loci (QTLs) associated with seed weight have been reported in the 11 bean chromosomes (Arriagada et al., 2022). However, most of the Pv01, Pv09, and Pv10 regions did not show variation within the Fabada marker class (Figure S5), whereas high concentrations of SNPs were observed in regions of the chromosomes Pv04, Pv05, Pv06, Pv07, Pv08, and Pv11. Many QTL for seed weight were located on chromosomes Pv01, Pv09, and Pv10 (Blair & Izquierdo, 2012; González et al., 2016; Sandhu et al., 2018). These findings suggest that chromosomal regions without SNPs have relevant roles in controlling the Fabada seed phenotype. In contrast, SNP‐rich regions do not have relevant roles in the genetic control of specific seed traits for this market class. In fact, in the chromosomes Pv04, Pv05, and Pv11, no major QTL for seed weight has been mapped in biparental populations having a Fabada parent (Murube et al., 2020). Nevertheless, the role of this type of variation should be confirmed in future studies using high‐throughput phenotyping of quantitative seed traits.

## 5 CONCLUSION

The genotyping of materials classified as Fabada showed a wide genotypic variation in this market class, suggesting that it is a landrace group rather than varieties derived from breeding programs. Genotyping also revealed redundant lines and homonymies among the accessions preserved in the SERIDA collection. Genetic erosion was detected when comparing diversity levels between the accessions preserved in the SERIDA collection and those collected during 2021. Most of the genetic diversity was maintained in the SERIDA collection, although some genotypes were not present. The observed erosion cannot be attributed to the diffusion of modern cultivars. In fact, these modern lines have higher genetic diversity levels than the currently cultivated lines. Therefore, their use could increase the variability within this market class.

## AUTHOR CONTRIBUTIONS

**Maria Jurado**: Formal analysis; investigation; software; writing—original draft. **Carmen Garcia‐Fernández**: Investigation; writing—review and editing. **Ana Campa**: Data curation; formal analysis; methodology; writing—review and editing. **Juan Jose Ferreira**: Conceptualization; funding acquisition; methodology; supervision; writing—original draft; writing—review and editing.

## CONFLICT OF INTEREST STATEMENT

The authors declare no conflicts of interest.

## Supporting information

Supporting Information

Supporting Information

Supporting Information

## Data Availability

The genotyping data supporting this study are available at the Dryad repository (https://doi.org/10.5061/dryad.djh9w0w5d). An image with the seeds of each line included in the panel was deposited at the Zenodo repository (https://doi.org/10.5281/zenodo.7015279; https://zenodo.org/record/7015279#.Y1veBORByUk)
