# Supplementary material for: Genetic erosion within the Fabada dry bean market class revealed by high‐throughput genotyping
Source: Plant Genome. 2023 Sep 19;16(4):e20379. doi: 10.1002/tpg2.20379 (PMC12807360; doi:10.1002/tpg2.20379)
Supplement: Supplementary file 1 [file TPG2-16-e20379-s001.pdf]

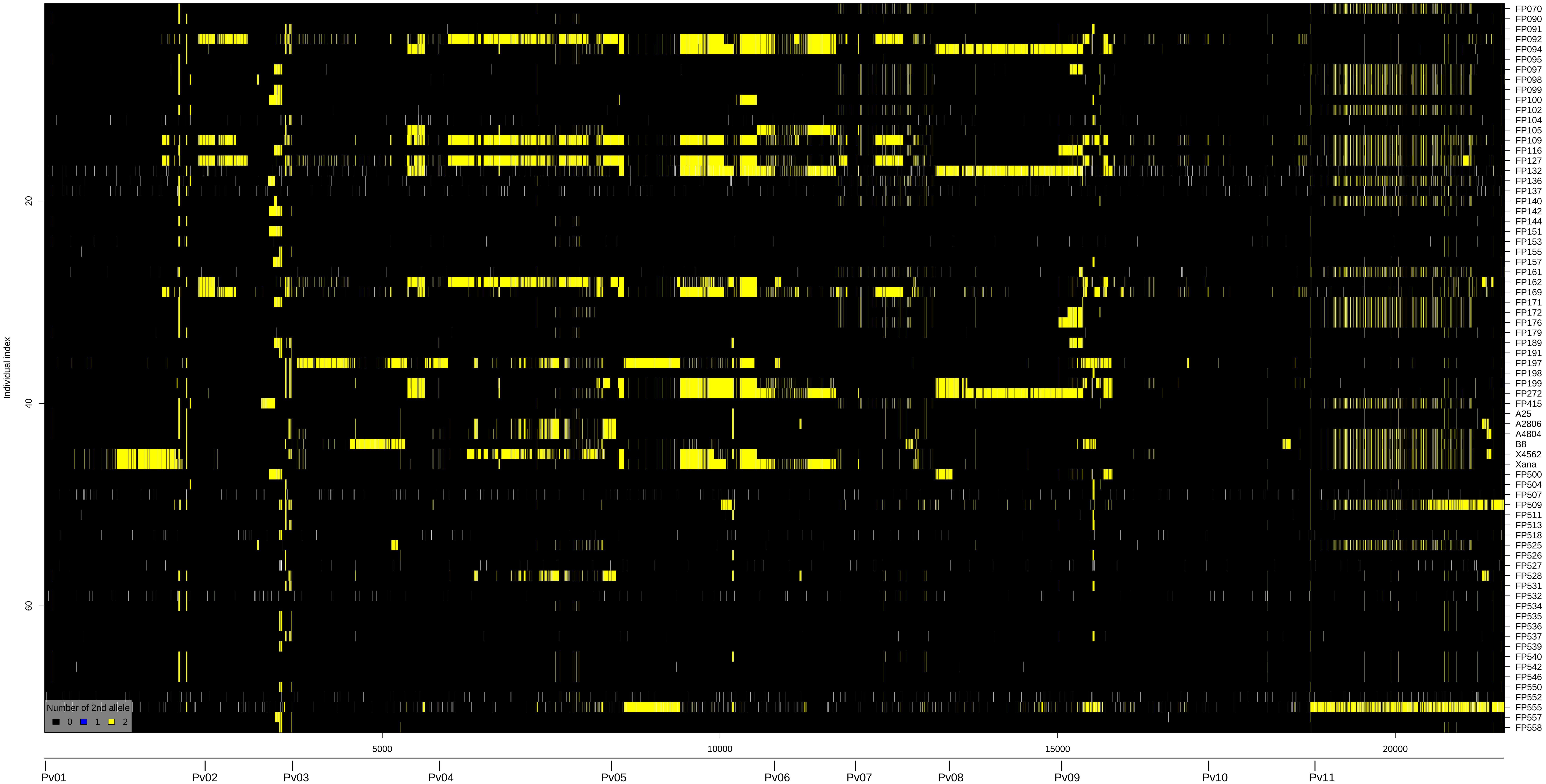
Individual index
20
40
60
FP070
FP090
FP091
FP092
FP094
FP095
FP097
FP098
FP099
FP100
FP102
FP104
FP105
FP109
FP116
FP127
FP132
FP136
FP137
FP140
FP142
FP144
FP151
FP153
FP155
FP157
FP161
FP162
FP169
FP171
FP172
FP176
FP179
FP189
FP191
FP197
FP198
FP199
FP272
FP415
A25
A2806
A4804
B8
X4562
Xana
FP500
FP504
FP507
FP509
FP511
FP513
FP518
FP525
FP526
FP527
FP528
FP531
FP532
FP534
FP535
FP536
FP537
FP539
FP540
FP542
FP546
FP550
FP552
FP555
FP557
FP558
Number of 2nd allele
0
1
2
5000
10000
15000
20000
Pv01
Pv02
Pv03
Pv04
Pv05
Pv06
Pv07
Pv08
Pv09
Pv10
Pv11
