# Supplementary material for: Genetic erosion within the Fabada dry bean market class revealed by high‐throughput genotyping
Source: Plant Genome. 2023 Sep 19;16(4):e20379. doi: 10.1002/tpg2.20379 (PMC12807360; doi:10.1002/tpg2.20379)
Supplement: Supplementary file 2 [file TPG2-16-e20379-s003.pdf]

Supporting Information

Supplemental Table S1.xls: Supplemental Table S1: List of the bean lines included in the FabaPanel with the passport data. The lines that conform to the different subsets to carry out the diversity analysis are indicated.

See excel file

Supplemental Table S2. Estimations of Nei´s genetic distances and Fst fixation index among the four populations (Conserved, Cultivar, Cultivated, Reference) using the package SambaR in the filtered FabaPanel population (107)

Fst index

	Conserved	Cultivar	Cultivated	Reference
**Conserved**		0.088	0.052	0.141
**Cultivar**			0.058	0.169
**Cultivated**				0.329
**Reference**				

Nei´s genetic distance

	Conserved	Cultivar	Cultivated	Reference
**Conserved**				
**Cultivar**	0.029			
**Cultivated**	0.031	0.028		
**Reference**	0.211	0.28	0.31	

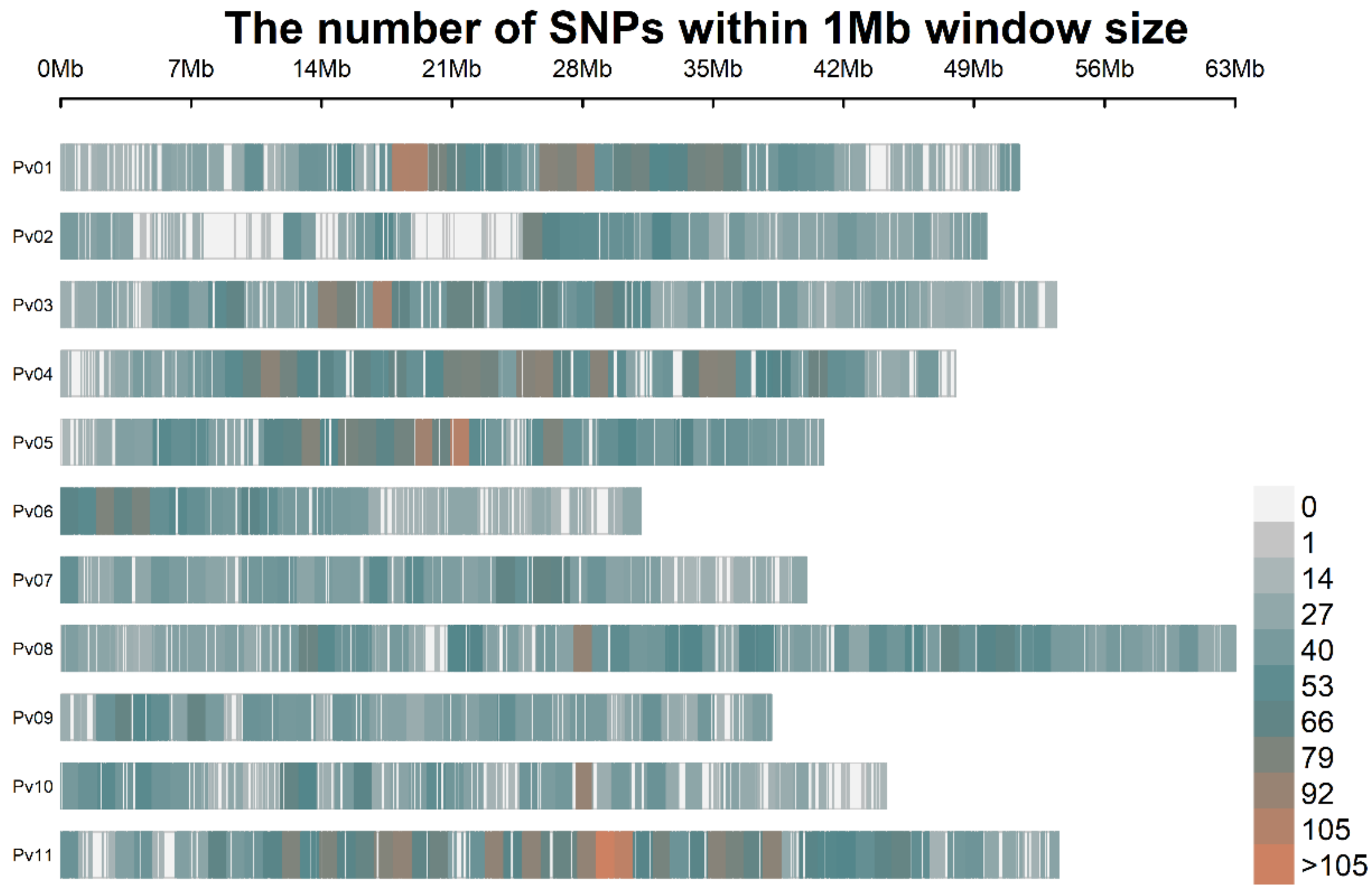

Figure S1. Distribution along the 11 bean chromosomes of the 22,259 SNP with which the FilterFabPanel1 was genotyped after filtering.

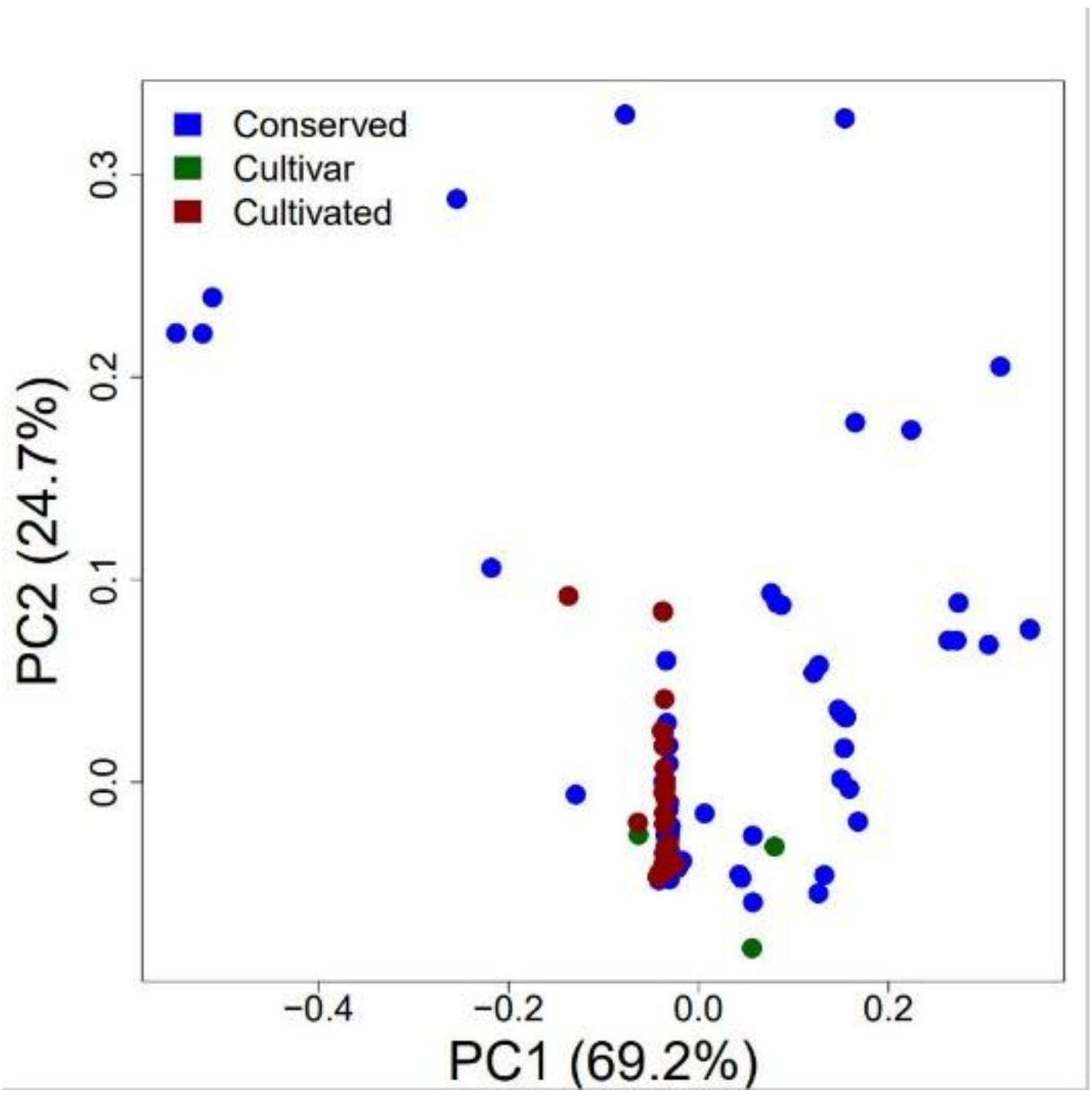

Supplemental Figure S2. Scatter plot obtained with the two mains coordinates reveled by the Principal Coordinate Analysis (PCoA) based on Hamming's genetic distance of the 159 lines classified as Fabada market class and genotyped with 21837 SNP

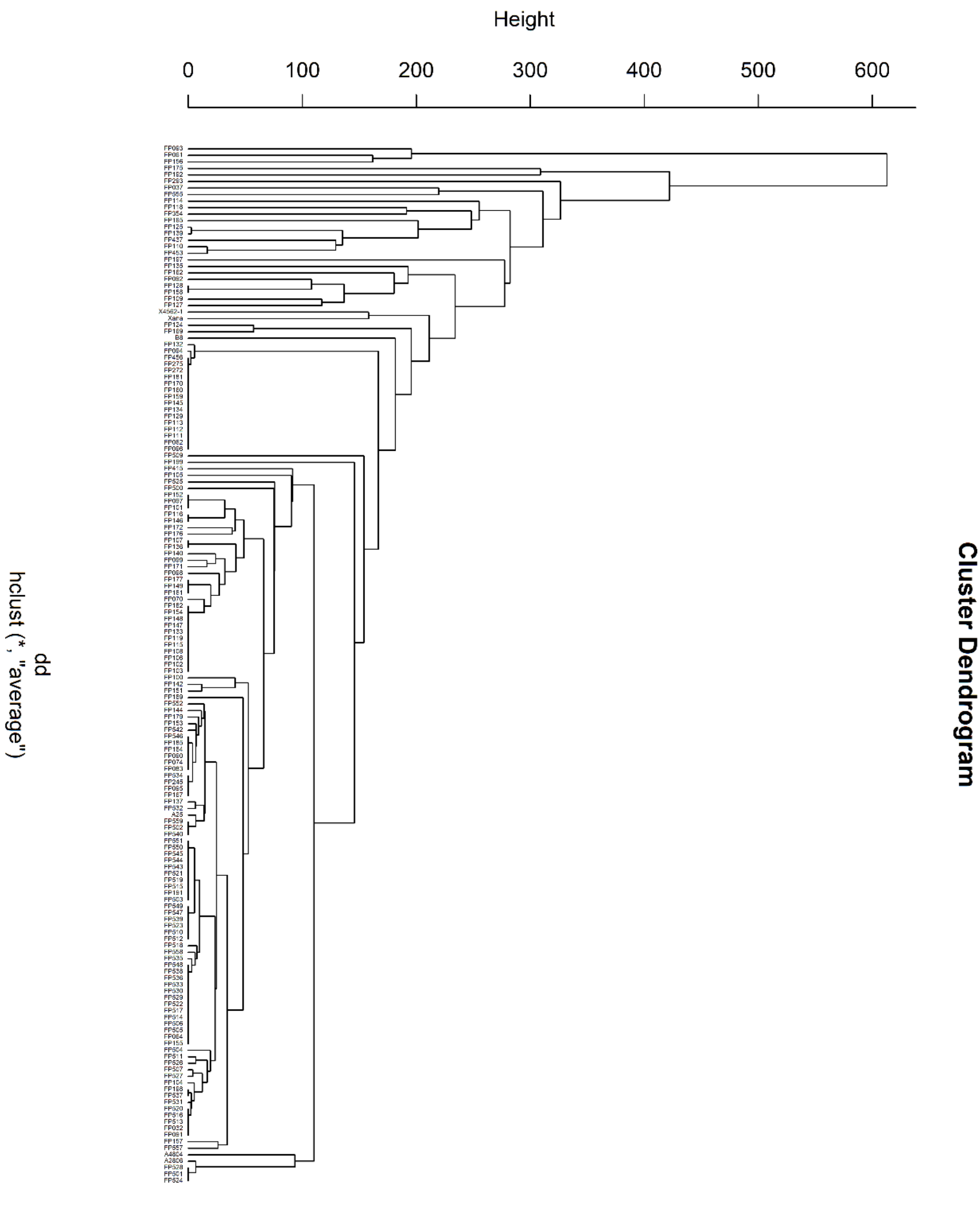

Supplemental Figure S3. Dendrogram obtained from FilterFabaPanel2 (159 lines recorded as Fabada market class) genotyped with 21837 SNP using the Euclidea distance and the UPGMA method.

a.

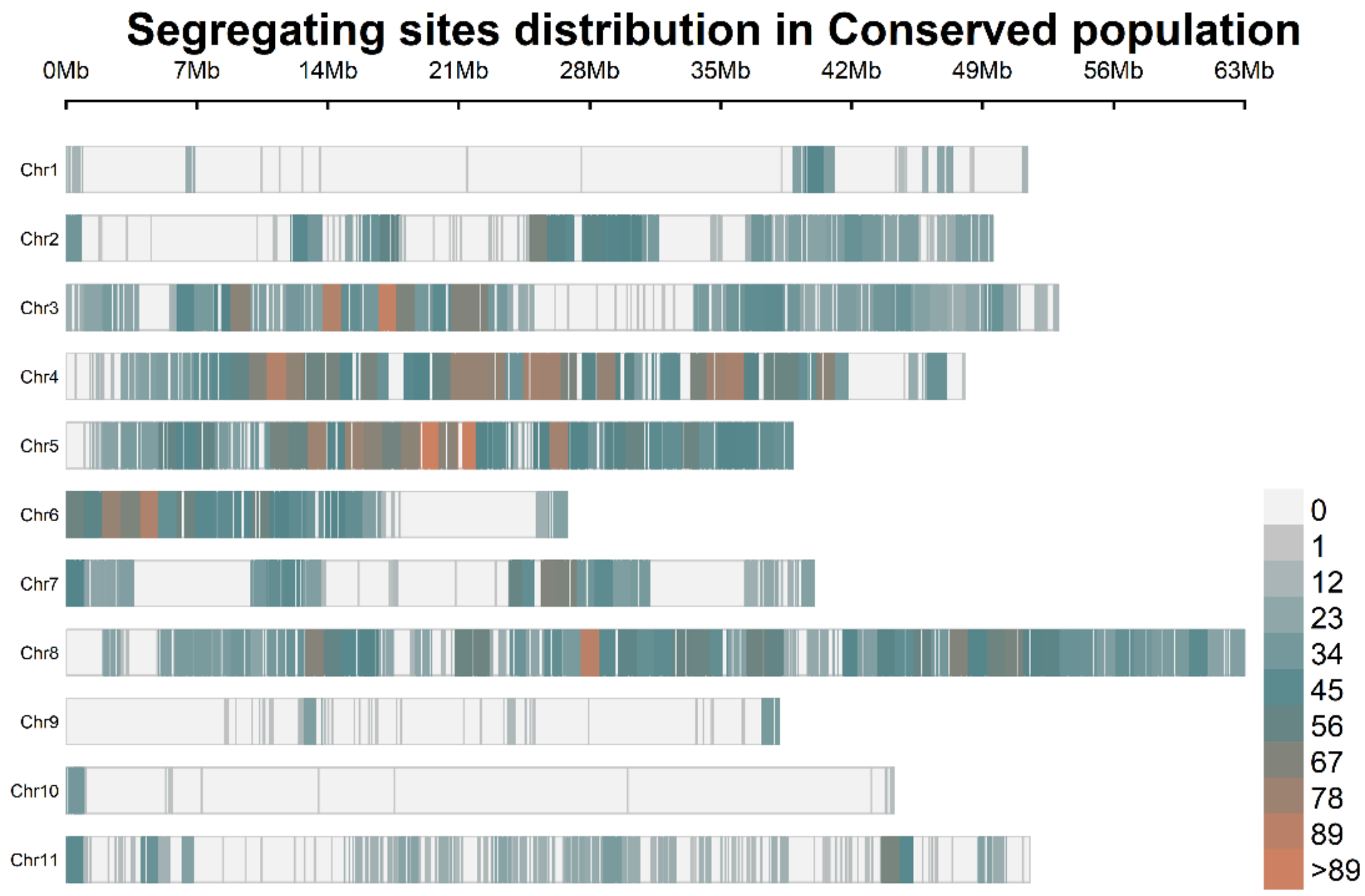
Segregating sites distribution in Conserved population
0Mb
7Mb
14Mb
21Mb
28Mb
35Mb
42Mb
49Mb
56Mb
63Mb
Chr1
Chr2
Chr3
Chr4
Chr5
Chr6
Chr7
Chr8
Chr9
Chr10
Chr11
0
1
12
23
34
45
56
67
78
89
>89

b.

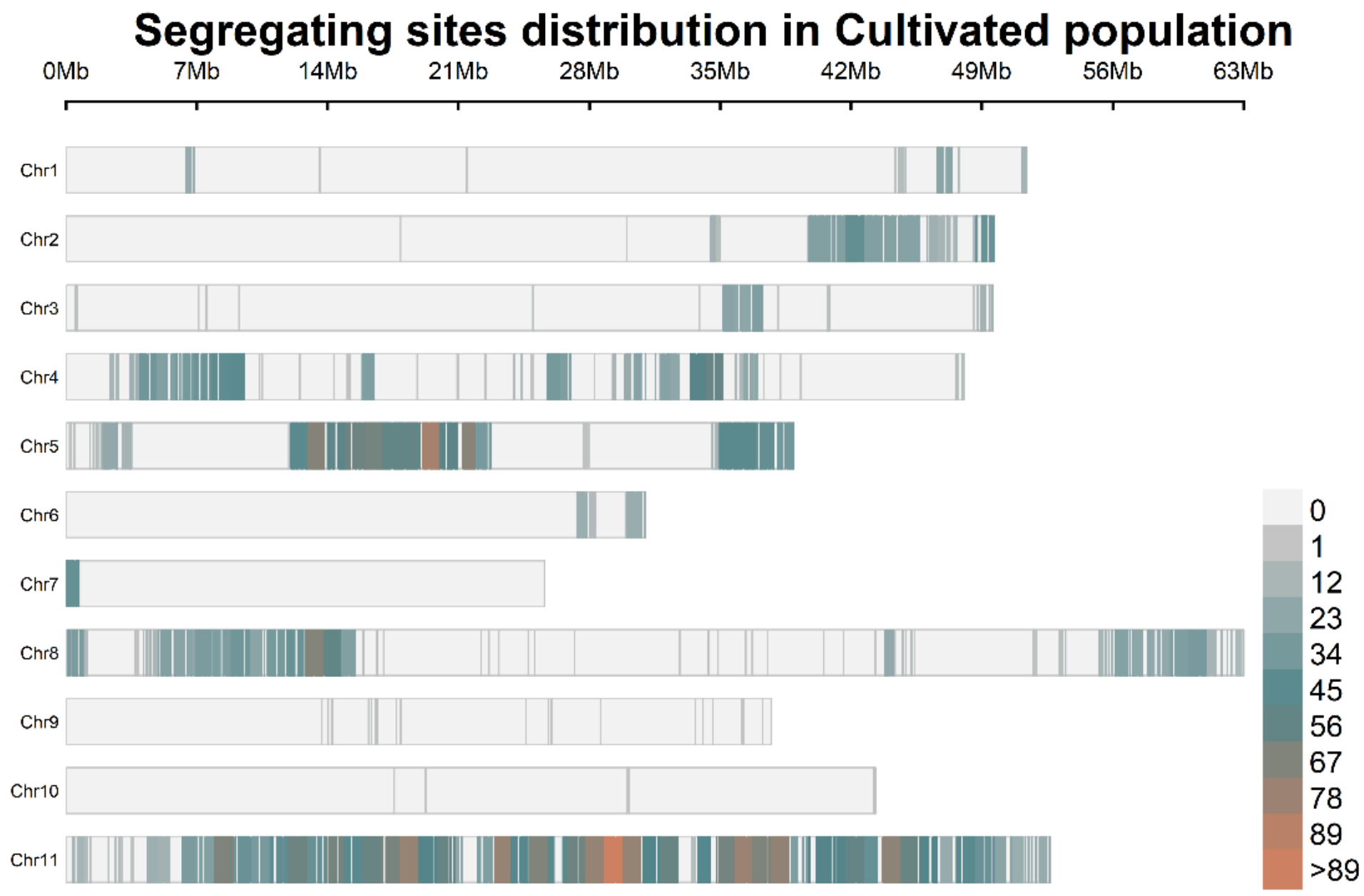
Segregating sites distribution in Cultivated population
0Mb
7Mb
14Mb
21Mb
28Mb
35Mb
42Mb
49Mb
56Mb
63Mb
Chr1
Chr2
Chr3
Chr4
Chr5
Chr6
Chr7
Chr8
Chr9
Chr10
Chr11
0
1
12
23
34
45
56
67
78
89
>89

c.

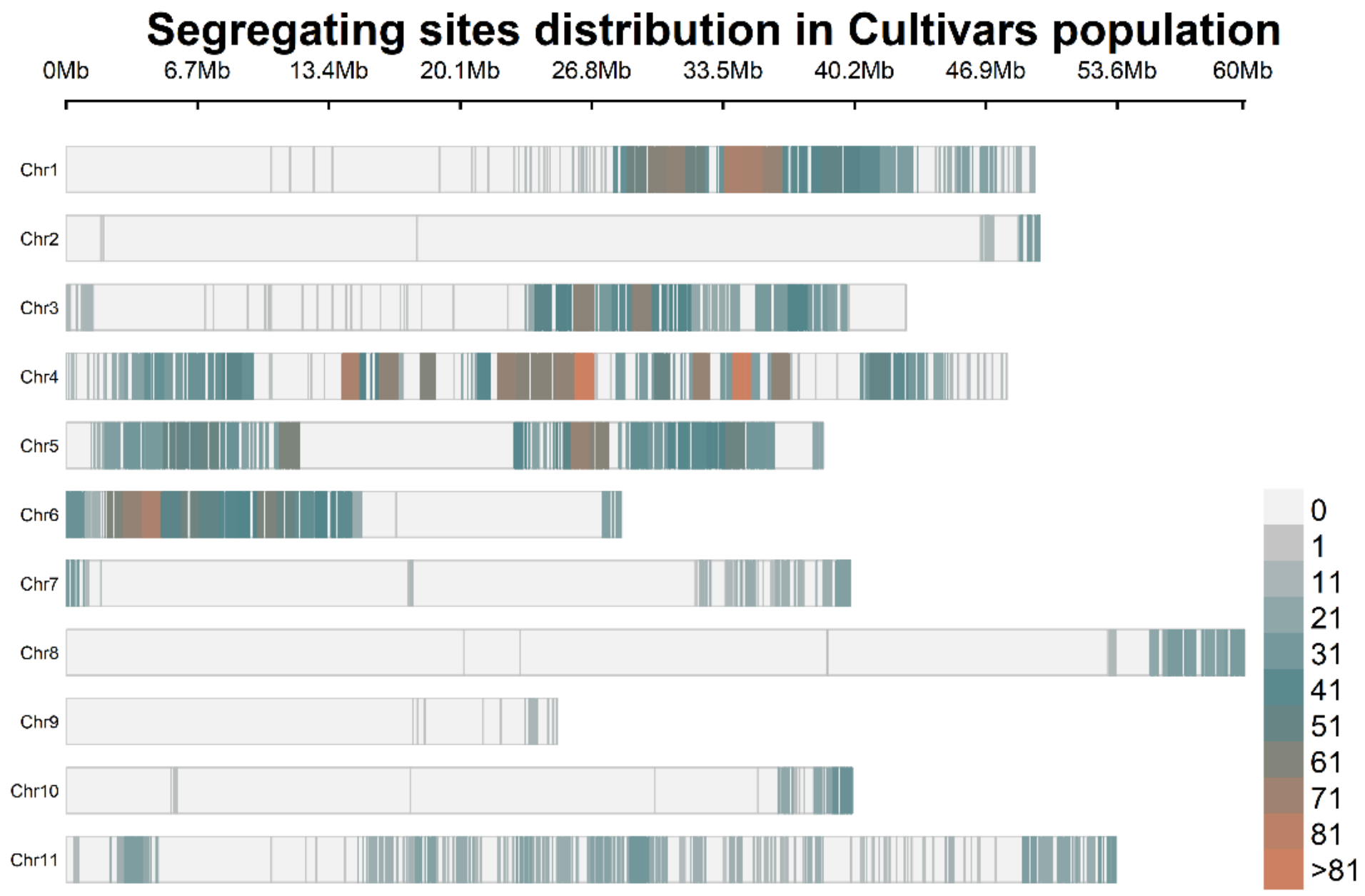

Supplemental Figure S4. Segregating site distributions per population along the 11 bean chromosomes. a) Conserved population. b) Cultivated population. c) Cultivar population

Supplemental Figure S5: Distribution of segregating sites (SNP) per bean chromosome and genotypes of the FilterFabaPanel3. Black, major allele; yellow, minor allele.

See pdf document
